# 100-Gbps per-channel all-optical wavelength conversion without pre-amplifiers based on an integrated nanophotonic platform

**DOI:** 10.1515/nanoph-2023-0264

**Published:** 2023-07-17

**Authors:** Ping Zhao, Zonglong He, Vijay Shekhawat, Magnus Karlsson, Peter A. Andrekson

**Affiliations:** Photonics Laboratory, Department of Microtechnology and Nanoscience, Chalmers University of Technology, 41296 Gothenburg, Sweden

**Keywords:** coherent optical communications, four-wave mixing, integrated waveguide, wavelength conversion

## Abstract

All-optical wavelength conversion based on four-wave mixing attracts intense interest in many areas, especially in optical fiber communications, due to the advantages of femtosecond response, modulation-format transparency, and high flexibility in optical network management. In this paper, we present the first optical translation of 32-GBaud 16QAM signals with an integrated Si_3_N_4_ nonlinear nanophotonic waveguide. An on-chip continuous-wave conversion efficiency of up to −0.6 dB from S band to C band is achieved in the dispersion-engineered low-loss Si_3_N_4_ nonlinear waveguide that is back-end compatible with complementary metal–oxide–semiconductor processes. The high conversion efficiency avoids the use of external optical amplifiers for signal demodulation. The converted idler is successfully received with a sensitivity penalty of less than 0.5 dB. Moreover, pre-amplifier-free multichannel wavelength conversion of over-100-Gbps coherent signals in C band is also demonstrated using the same Si_3_N_4_ nanophotonic waveguide via changing the pump wavelength, which shows good flexibility in all-optical signal processing. Additionally, wavelength conversion with a bandwidth over 100 nm can be expected by optimizing the current Si_3_N_4_ nanophotonic waveguide, which is promising for commercial coherent fiber communications and has bright prospects in various areas including optical signal processing, imaging, optical spectroscopy, and quantum optics.

## 1 Introduction

Global Internet traffic has been growing fast, leading to a strong desire for high-capacity and flexible optical fiber communications (OFC), especially coherent OFC which exhibits speeds over 100 Gbps per channel with high spectral efficiencies (SEs) as well as long reach. Wavelength conversion (WC) in the optical domain has long been pursued in OFC, which enables all-optical reconfigurability of wavelength-division-multiplexing networks [1]. In addition, WC can mitigate optical fiber nonlinearities for telecommunication and is promising in commercial coherent OFC which dominates the optical communication market [2, 3]. Another potential of WC is to reuse the mature transmitters, receivers, and erbium-doped fiber amplifiers (EDFAs), to realize optical transmission beyond C and L bands [4, 5], since more and more wavelength bands are being considered for OFC to catch up with the trend of network traffic growth [6]. WC with high conversion efficiencies (CEs, the power ratio of generated idler to the input signal) is important to OFC because the optical amplification of the idler at the receiver can be avoided. Previous studies show that transparent WC for OFC can be achieved using nonlinear silica fibers [7–9]. However, hundreds of meters of fibers were required and exhibited limited dispersion-engineering abilities for broadband operation, disturbed by polarization drifts. Due to the advantages of robustness, compactness, and excellent dispersion engineering, WC based on a single pump source and integrated nonlinear waveguides is attractive, which has been demonstrated in both *χ*^(2)^ and *χ*^(3)^ platforms. Bulk periodically-poled lithium niobate (PPLN) waveguides have shown high CEs for high-speed WC [10]. Nevertheless, the fabrication of low-loss thick PPLN waveguides with precise micromachining [11], is challenging. Recently, thin-film (TF) PPLN nanophotonic waveguides fabricated via etching have also been investigated for the WC of high-speed coherent optical signals [12], while the losses of such waveguides were high and resulted in low CEs. Generally, periodic poling is required for *χ*^(2)^ platforms, which requires a fixed pump wavelength and limits the utilization of WC in WDM networks. To translate a wavelength-fixed signal to a desired idler channel, another *χ*^(2)^-waveguide needs to be fabricated. Four-wave-mixing (FWM) based high-speed WC in *χ*^(3)^ integrated nanophotonic waveguides are intensively studied, enabling tunability of both signal and pump wavelengths and being transparent to modulation format. In early demonstrations, active III–V waveguides were utilized for WC due to high nonlinearities [13], while it was costly and difficult to fabricate these waveguides which also had a slow temporal response as a result of long carrier recovery time [14, 15]. Consequently, dielectric *χ*^(3)^ integrated nanophotonic waveguides are preferred for high-speed WC. So far, various kinds of dielectric *χ*^(3)^ materials with large nonlinear coefficients (*γ*), such as chalcogenide glass [16], pure/p-i-n silicon [17–20], silicon germanium [21], Hydex glass [22], and aluminum gallium arsenide (AlGaAs) [5, 23], have been investigated for high-bit-rate waveguide-based WC. However, additional optical amplification (AOA) of idler waves generated in these *χ*^(3)^ waveguides was required, due to low CEs limited by low continuous-wave (CW) power handling ability or high nonlinear-optical-absorption/coupling loss.

In this paper, we experimentally demonstrate flexible 100-Gbps all-optical WC of single-polarization coherent signals using a low-loss dispersion-engineered Si_3_N_4_ nonlinear nanophotonic waveguide which is free of two-photon absorption and exhibits excellent power handling ability. The modulation format is 16 quadrature amplitude modulation (QAM) with a 32-GBaud rate, corresponding to a line rate of 128 Gbps. AOA-free WC for both signal and idler channels is realized with a *χ*^(3)^ integrated waveguide for the first time, benefitting from a high on-chip/fiber-to-fiber (F2F) CE of up to −0.6/−5.6 dB. Besides, pump tuning, as well as multichannel processing of the optical spectral translation with the same Si_3_N_4_ integrated nonlinear waveguide, is also successfully implemented, which indicates that the Si_3_N_4_-waveguide-based WC is promising for high-speed coherent OFC links and networks.

## 2 Experiments and results

### 2.1 Low-loss dispersion-engineered Si_3_N_4_ nonlinear nanophotonic waveguide

**Figure 1:**
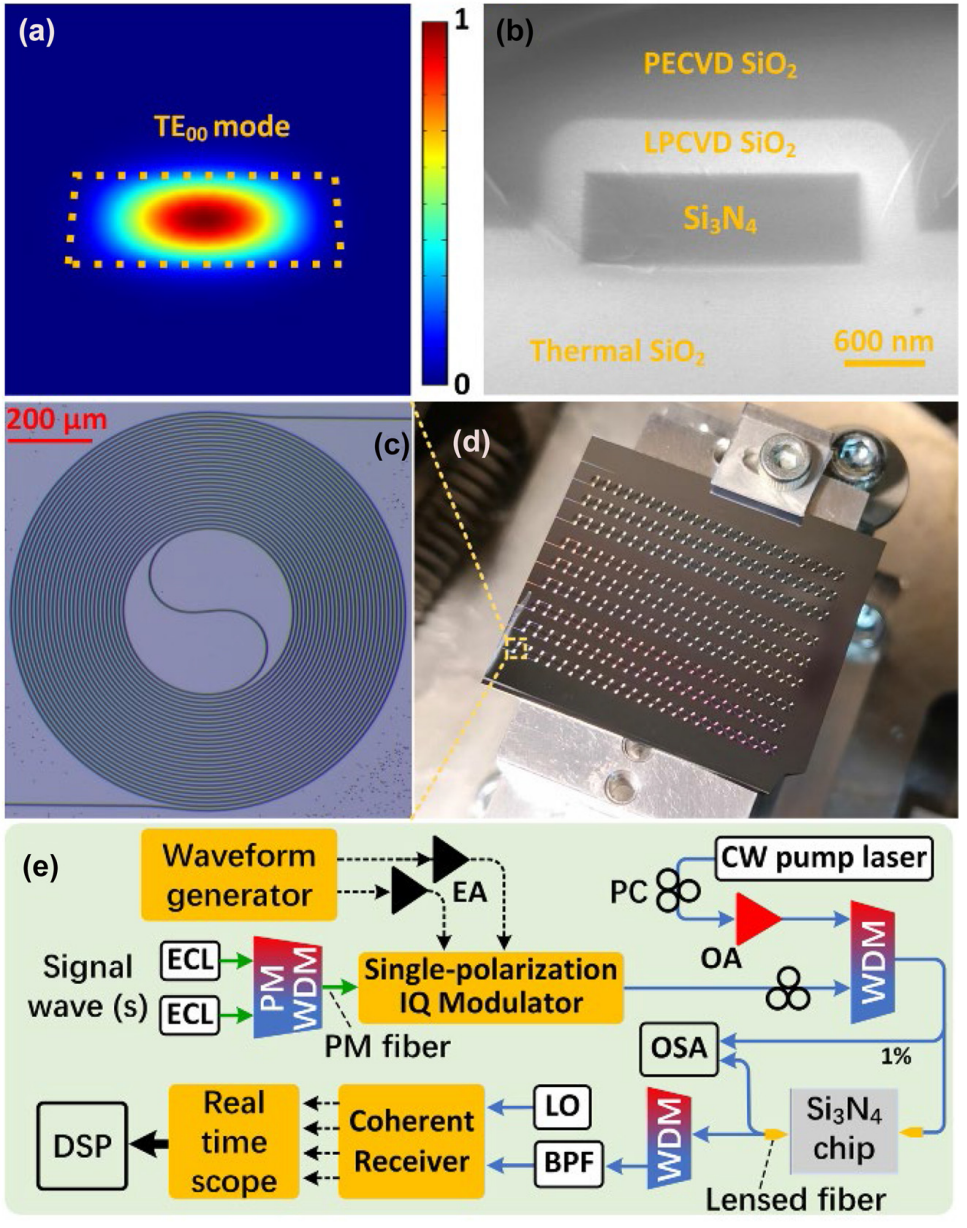
Experimental setup of wavelength conversion based on low-loss dispersion-engineered Si_3_N_4_ nonlinear nanophotonic waveguides. (a) Simulated intensity profile of the fundamental transverse electric (TE_00_) mode of a low-loss high-confinement Si_3_N_4_ waveguide. The dotted box is the boundary of the Si_3_N_4_ core which is 690 nm high and 1950 nm wide and leads to anomalous dispersion at 1550 nm. (b) Scanning electron microscope image of the cross section of a manually-cleaved nonlinear Si_3_N_4_ waveguide fabricated. This Si_3_N_4_ waveguide is 1.42 m and shaped into 23 cascaded spirals with a chip area of about 25 mm^2^, due to the limited writing field of 1 × 1 mm^2^ in electron-beam lithography the image blurs slightly due to the low conductivity of the sample without metal coating. LPCVD, low pressure chemical vapor deposition; PECVD, plasma-enhanced chemical vapor deposition. (c) Optical microscope image of a spiral unit of the Si_3_N_4_ waveguide. The minimum radius in the spiral waveguide is 200 μm. (d) Picture of a chip with 9 nonlinear Si_3_N_4_ waveguides with the same design. The chip was manually cleaved. (e) Experimental setup of the all-optical wavelength conversion of 32-GBaud 16QAM signals with the nonlinear Si_3_N_4_ waveguide. CW, continuous-wave; PM, polarization-maintaining; PC, polarization controller; IQ, in-phase and quadrature; ECL, external-cavity laser; WDM, wavelength-division multiplexer; OSA, optical spectrum analyzer; BPF, band-pass filer; LO, local oscillator; DSP, digital signal processing. One percent of the optical power at the input and output lensed fibers coupled to the Si_3_N_4_ chip is tapped for spectrum monitoring. The dashed and solid lines are electrical and optical links, respectively.

In principle, the phase mismatch parameter (*κ*) of FWM needs to be kept small to achieve a high CE for single-pump WC, which can be approximately described as *κ* = *γP* + *β*_2_Δ*ω*^2^/2 with *P*, *β*_2,_ and Δ*ω* to be the pump power, group velocity dispersion (GVD) at the pump wavelength and frequency difference between the pump and signal wavelengths [24, 25]. Accordingly, anomalous GVD (*β*_2_ < 0) can facilitate reducing the phase mismatch parameter, increasing the CE, and improving the WC performance. Distinct from many nonlinear integrated nanophotonic waveguides, a Si_3_N_4_ nonlinear waveguide can support Watt-level CW optical laser power and is an excellent candidate for time-continuous optical signal processors [26]. Figure 1(a) shows the simulated intensity profile of the fundamental transverse electric (TE_00_) mode of the Si_3_N_4_ nonlinear waveguide we designed. The Si_3_N_4_ core is 690 nm high and 1950 nm wide with a side wall angle of 87°. The 690 nm thickness was chosen to tolerate GVD change which may be caused by thickness, width and radius variations along the meter-long Si_3_N_4_ nonlinear waveguide. The dotted box is the boundary of the Si_3_N_4_ core. The theoretical GVD and nonlinear coefficient of the straight Si_3_N_4_ waveguide at 1550 nm are −28 ps^2^/km and 0.96 (Wm)^−1^, respectively. As can be seen in Figure 1(a), the TE_00_ mode almost vanishes at the waveguide side walls, which helps to reduce scattering loss. Figure 1(b) presents the scanning electron microscope image of the cross-section of a manually-cleaved nonlinear Si_3_N_4_ waveguide fabricated with a subtractive process [27]. Multiple-pass electron-beam lithography (EBL), one key fabrication process, was used to reduce the side wall roughness of Si_3_N_4_ waveguides [28]. The bottom silica cladding was formed by the thermal oxidization of a silicon wafer. Low-pressure chemical vapor deposition (LPCVD) was used to generate a dense silica top cladding with a thickness of 400 nm on the Si_3_N_4_ waveguide. Finally, another 2-μm-thick silica layer was used to increase the robustness of the Si_3_N_4_ chip, generated by fast plasma-enhanced chemical vapor deposition (PECVD). The fabricated Si_3_N_4_ nanophotonic waveguides are 1.42 m long. Since the writing field of EBL in our cleanroom was only 1 × 1 mm^2^, we shaped the meter-long Si_3_N_4_ waveguide into 23 cascaded spirals along one direction to obtain small footprints with chip areas of about 25 mm^2^ each. Careful calibration was performed to minimize the stitching errors between adjacent spirals before the fabrication of the meter-long Si_3_N_4_ waveguide. Figure 1(c) depicts the optical microscope image of a spiral unit of the nonlinear Si_3_N_4_ waveguide. The minimum spiral radius is 200 μm, leading to negligible bending loss. As any defect in the waveguide may be generated during fabrication and would cause large optical loss, multiple Si_3_N_4_ nanophotonic waveguides with the same design were manufactured in our cleanroom to increase the success rate of the device. Figure 1(d) presents the picture of a Si_3_N_4_ chip with nine 1.4-m-long nonlinear Si_3_N_4_ waveguides of which the lowest propagation loss is 1.4 dB/m. Manual cleaving was applied to the chip for edge coupling. Optical frequency domain reflectometry was utilized to measure the waveguide propagation loss [29]. Failure of several meter-long Si_3_N_4_ nonlinear waveguides was observed, as a result of the waveguide defect. To realize efficient coupling between the nanophotonic Si_3_N_4_ chip and lensed fibers, the waveguide width was adiabatically tapered down to 300 nm with a taper length of 400 μm for the TE_00_ mode of the Si_3_N_4_ waveguide. The average fiber-to-chip coupling loss was 2.5 dB/facet, which was estimated by considering the propagation loss. The ultra-low propagation loss tegother with the meter-scale length, large nonlinear coefficient and excellent CW power handling ability of the Si_3_N_4_ nanophotonic waveguides paves way to high WC CEs [30].

### 2.2 Wavelength conversion of high-speed coherent optical signals

Figure 1(c) shows the WC experimental setup for spectral translation from S Band to C band, based on the fabricated nonlinear Si_3_N_4_ waveguide. Two electrical signals from a programmable waveform generator were amplified and fed to a single-polarization in-phase and quadrature (IQ) modulator to generate a 32-GBaud 16QAM optical signal. The signal carrier was a CW external-cavity laser (ECL) with a power of 13 dBm. Another low-noise CW laser, i.e., pump at 1536.7 nm, was amplified and combined with the signal via a wavelength-division multiplexer (WDM). Two lensed fibers were used to couple the light into/out of the Si_3_N_4_ chip. The polarization states of the signal and pump were aligned to the TE_00_ mode via polarization controllers (PCs), respectively. The effective input pump power on the TE_00_ mode was about 30.9 dBm, considering the fiber-to-chip coupling loss. The signal laser was set to be 1528 nm, near the phase-matching wavelength in S band, and created an idler wave at 1545.6 nm in C band. We used another WDM to block the pump from the signal and idler waves at the Si_3_N_4_ waveguide output. One percent of the optical field at input and output lensed fibers was tapped and recorded by an optical spectrum analyzer (OSA), respectively. The optical signal and idler after WC were filtered by a tuneable band-pass filter (BPF) and separately detected by a coherent receiver with a tunable local oscillator (LO). The LO power was 9 dBm. The electrical signals after the coherent receiver were recorded by a real-time scope and processed by offline digital signal processing (DSP) algorithms for data recovery and the calculation of bit-error rate (BER) [31]. Note that no optical amplification in the signal path from the transmitter to the receiver was used. For the application of S-to-C-band WC, one signal laser was used since we only had one available ECL covering S band. Moreover, we demonstrated flexible and multichannel WC in C band with the same nonlinear Si_3_N_4_ waveguide, by adjusting the pump wavelength to 1545.7 nm and changing the signal wavelengths accordingly. The on-chip pump power was kept the same as in the previous experiment. Two CW signal lasers with wavelengths at 1552.5 nm and 1554.1 nm were combined by a polarization-maintaining WDM and sent into the IQ modulator to generate two 32-GBaud 16QAM optical signals, which were simultaneously converted to two short wavelengths by the integrated nonlinear Si_3_N_4_ nanophotonic waveguide.

**Figure 2:**
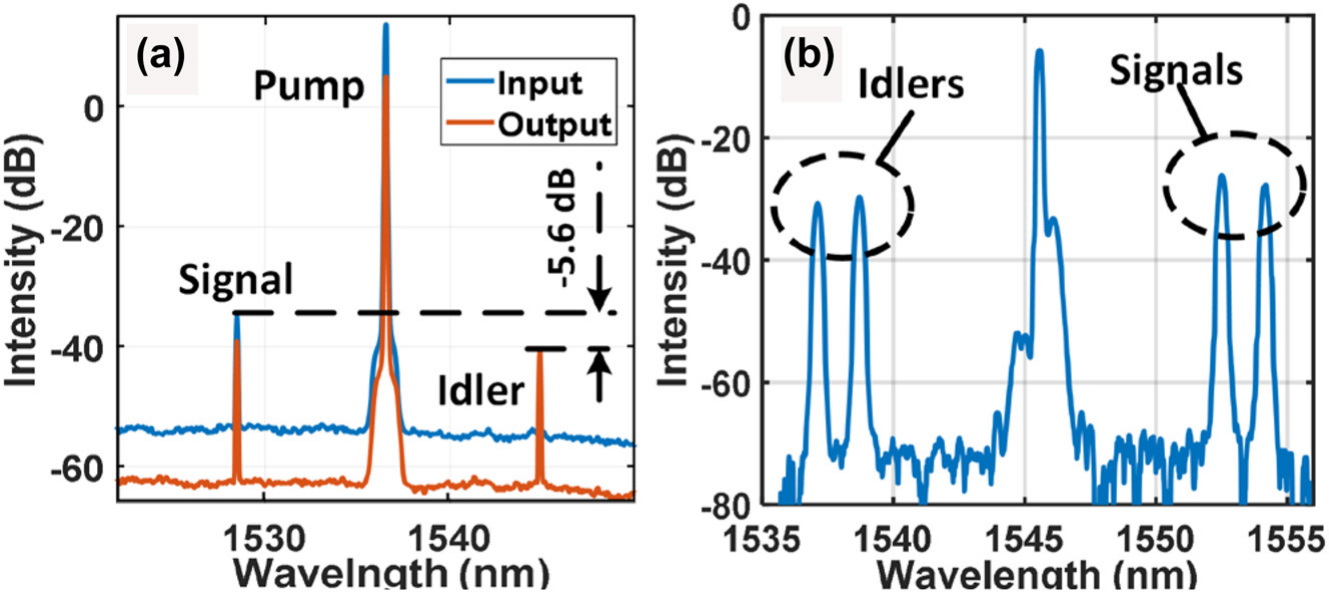
Optical spectra of 32-GBaud 16QAM signals after wavelength conversion. (a) Spectra monitored at the ports of the input and output lensed fibers coupled to the Si_3_N_4_ waveguide in the case of single-channel wavelength conversion from S band to C band. The pump wavelength is 1536.7 nm. The monitoring devices are two 1 % optical fiber couplers. (b) Dual-channel wavelength-conversion spectrum recorded at the output signal port of the WDM after the Si_3_N_4_ waveguide pumped at 1545.7 nm.

Figure 2(a) shows the spectra at the Si_3_N_4_ waveguide input (blue) and output (red) lensed fibers, monitored via two 20 dB couplers respectively. A F2F CE of −5.6 dB is obtained at the idler wavelength of 1545.6 nm, as can be seen in Figure 2(a). A corresponding on-chip CE of −0.6 dB is estimated by considering the coupling loss. In addition, Figure 2(b) shows the WC spectrum recorded at the output signal port of the 1545.7 nm WDM following the output lensed fiber. Two idlers are simultaneously generated at 1538.7 nm and 1537.1 nm, with F2F CEs of −7 dB and −7.5 dB, respectively. The F2F CEs for the 1545.7 nm pump are lower than that in the case of 1536.7 nm pump. The pump power difference on the TE_00_ mode may contribute to the CE variation due to the wavelength-dependent loss of the current Si_3_N_4_ waveguide. As can be seen in Figure 2(b), no mixing term is visible between the adjacent signal/idler waves, indicating that the cross-talk between multiple channels is negligible. Moreover, the pump wavelength was flexibly adjusted without changing the nonlinear Si_3_N_4_ waveguide, which is an advantage over the *χ*^(2)^-based platforms.

**Figure 3:**
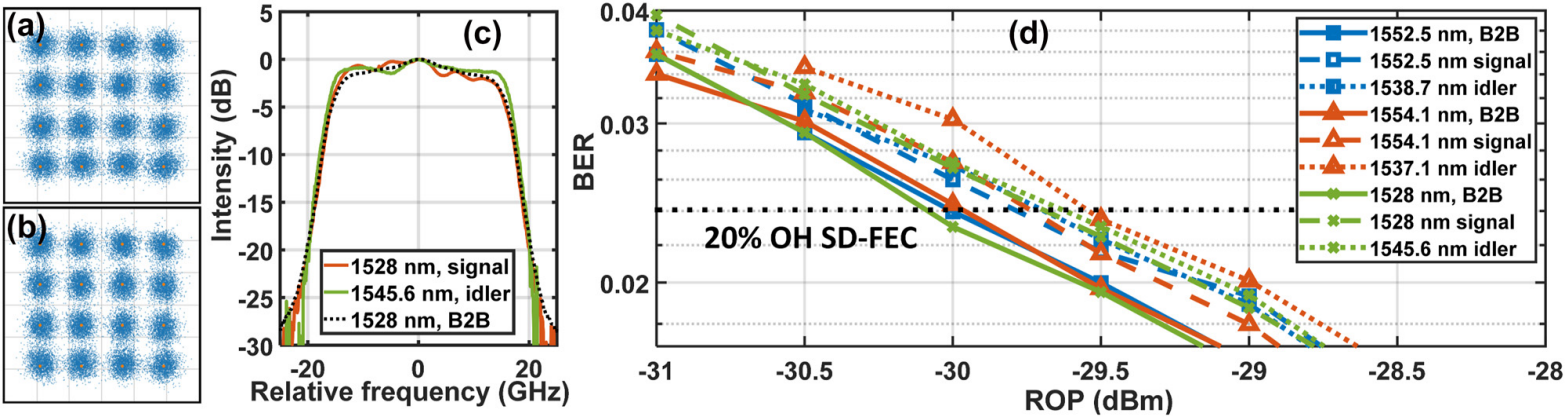
Characterization of signals after wavelength conversion. (a) 1528 nm signal and (b) corresponding 1545.6 nm idler constellation diagram recovered by the offline DSP algorithm. The pump wavelength was 1536.7 nm. (c) Optical spectra with frequency relative to the central carrier wavelengths at 1528 nm (red line) and 1545.6 nm (green line) for post wavelength conversion (solid lines) and back-to-back (B2B) 1528 nm signal (dotted line). (d) Bit error rate (BER) varying with received optical power (ROP) at different wavelength channels in the two pump cases. The solid, dashed and dotted lines are for B2B signals, post-chip signals and converted idlers, respectively. The black dotted line corresponds to a BER threshold of 0.024 for a typical soft-decision forward error correction with 20 % overhead in commercial coherent optical fiber communications.

Figure 3(a) and (b) show recovered constellation diagrams of the 1528 nm post-WC signal and the generated 1545.6 nm idler, respectively. The corresponding normalized optical spectra varying with the frequency relative to central carriers are presented by the red and green curves in Figure 3(c), respectively. The black-dotted line in Figure 3(c) is the spectrum of the back-to-back (B2B) 1528 nm signal. Minor spectral distortions during WC are observed as shown by Figure 3(c), which is believed to be due to weak multimode interference in the Si_3_N_4_ waveguide. The spectral characteristics of the post-WC 1552.5 nm and 1554.1 nm signals as well as 1538.7 nm and 1537.1 nm idlers are similar to that of the 1528 nm signal. Figure 3(d) depicts the BER versus received optical power (ROP) at different signal and idler wavelengths. We can see in Figure 3(d) that the penalties for the six post-WC wavelengths are within 0.5 dB, compared with the B2B cases at a BER threshold of 0.024 for typical 20 %-overhead (OH) soft-decision (SD) forward error correction (FEC) in commercial coherent OFC systems [32]. Table 1 summarizes state-of-the-art WC of single-polarization optical signals with high line rates (LRs) per channel based on integrated dielectric nonlinear nanophotonic waveguides. As can be seen, this work is the first to realize the signal/idler-AOA-free WC at a LR of 128 Gbps coherent signal with a net data rate over 100 Gbps in both *χ*^(2)^ and *χ*^(3)^ integrated nanophotonic platforms.

**Table 1:** Overview of single-pump FWM-based WC with integrated dielectric nonlinear nanophotonic waveguides for high-speed-per-channel optical fiber communications.

F2F CE (dB)	On-chip CE (dB)	Material	Modulation format	Line rate (Gbps)	Band	Idler	Year	Ref.
						amplification		
−25.6	−12.8	Chalcogenide	RZ-OOK	160	C	Needed	2010	[16]
−34.5	−31.5	Si	RZ-DPSK	640	C	Needed	2011	[17]
25.7	−21.5	Si_0.8_Ge_0.2_	40-GBaud QPSK	80	C	Needed	2013	[21]
−35	−17.5	Si	28-GBaud 16QAM	112	C	Needed	2014	[20]
−40	−29.5	Hydex-glass	32-GBaud QPSK	64	C	Needed	2016	[22]
−25.5	−9.5	p-i-n Si	32-GBaud 16QAM	128	C	Needed	2017	[18]
−17	−14.2	AlGaAs	10-GBaud 256QAM	70	C	Needed	2017	[23]
−19	−9.4	p-i-n Si	16-GBaud 16QAM	64	C	Needed	2019	[19]
−20	−10	TF-PPLN	23-GBaud 16QAM	92	C	Needed	2022	[12]
NA	−24.5	AlGaAs	32-GBaud 16QAM	128	C, 2-μm	Needed	2022	[5]
−5.6	−0.6	Si_3_N_4_	32-GBaud 16QAM	128	S, C	Not needed	2023	This work

## 3 Discussion

**Figure 4:**
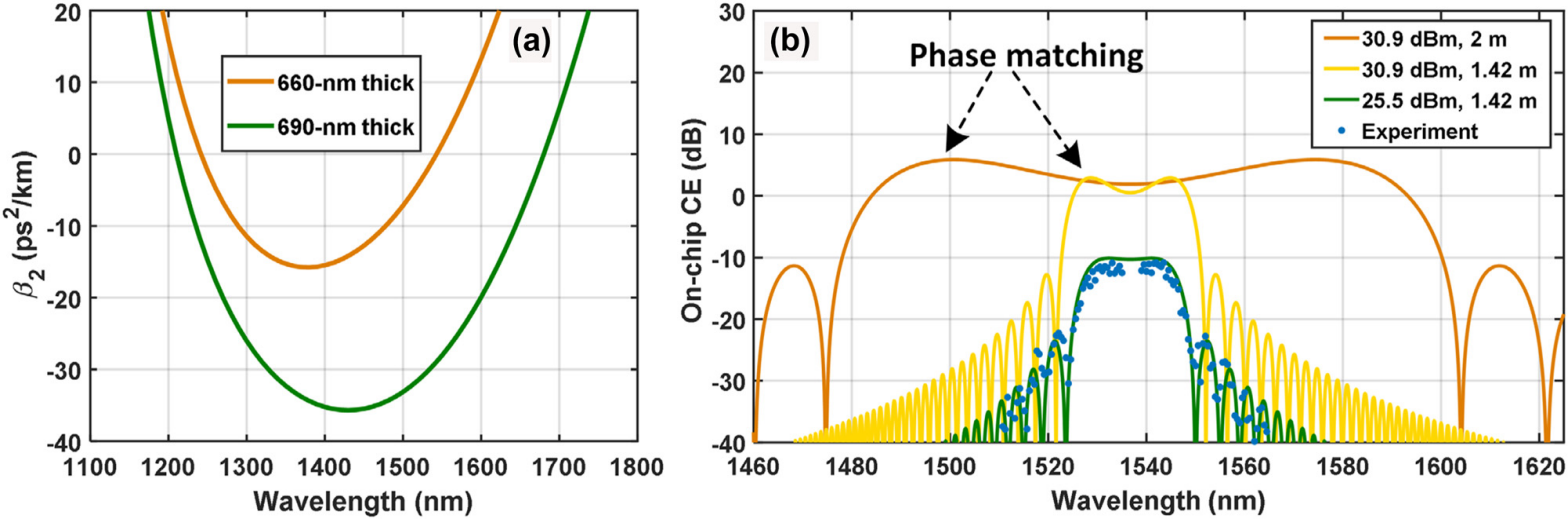
Dispersion profile of nonlinear Si_3_N_4_ waveguides and CE spectra. (a) Group velocity dispersion varying with wavelength for Si_3_N_4_ waveguides with thickness of 660 nm (orange, optimized) and 690 nm (green, used in the experiment), respectively. The width of the Si_3_N_4_ waveguide is 1950 nm. (b) Simulated (solid) and measured (blue dot) on-chip CE spectra for different nonlinear Si_3_N_4_ waveguides pumped at 1536.7 nm. The orange line corresponds to a 660 nm × 1950 nm Si_3_N_4_ waveguide with a length of 2 m and a pump power of 30.9 dBm. The green and yellow lines are for the cases of the 690 nm × 1950 nm Si_3_N_4_ waveguide used in the experiment with pump powers of 25.5 dBm and 30.9 dBm, respectively.

The bandwidth of the demonstrated WC is limited by the large GVD of the current Si_3_N_4_ waveguide of which the thickness was chosen for large fabrication tolerance. Figure 4(a) shows the calculated GVD spectral profiles of a 1950-nm-wide Si_3_N_4_ waveguide with two different thicknesses. By reducing the waveguide thickness from 690 nm (green) to 660 nm (orange), the GVD will decrease from −32 ps^2^/km to −2 ps^2^/km at 1536.7 nm wavelength, as shown by Figure 4(a). For such a small decrease in the Si_3_N_4_ waveguide thickness, the propagation loss as well as the nonlinear coefficient would change marginally. Hence, the WC bandwidth can be increased, and the CE would be retained. Figure 4(b) presents the measured (blue dot) and simulated (solid) spectra of on-chip CE with different pump powers and lengths, respectively. The green solid line corresponds to a pump power of 25.5 dBm. Such a relatively-low pump power helped to reduce the nonlinear term in the phase mismatch parameter and improve the GVD estimation of the Si_3_N_4_ waveguide based on the WC spectrum, via observing the dip wavelengths [33]. The experimental result agrees well with the theoretical one when a GVD of −45 ps^2^/km at 1536.7 nm wavelength was given in the simulation. The discrepancy between the designed and fitted GVD may be due to the variations of waveguide geometry parameters (width thickness and bending radius). Based on this fitted GVD, the theoretical bandwidth of the demonstrated WC for on-chip CE larger than −3 dB would be 27 nm, according to the yellow line in Figure 4(b). Besides, the theoretical on-chip CE at 1529 nm wavelength is 2.5 dB larger than at the wavelengths near the pump, which is attributed to the anomalous-GVD-lead phase matching.

By using a 660 nm × 1950 nm waveguide and increasing the waveguide length from 1.42 m to 2 m, one can expect a WC bandwidth of about 115 nm as illustrated by the orange curve in Figure 4(b) for the same pump power and waveguide loss in the experiments. Not only the decrease in GVD but also the nonlinear phase matching contributes to the increase of the WC bandwidth. It is inspiring that a theoretical on-chip CE of 6 dB at the phase-matching wavelength can be obtained with the increase in the Si_3_N_4_ waveguide length. This optimized Si_3_N_4_ waveguide geometry for broadband WC is feasible and is under development in our cleanroom. These geometrical features of the Si_3_N_4_ waveguide can also be realized in industrial nanofabrication foundries. Signle-polarization WC was performed in our experiments. To be adapted to commercial coherent optical fiber communications with polarization-diversity multiplexing, polarization-insensitive WC can also be implemented in the Si_3_N_4_ nanophotonic platform [34, 35].

With respect to the implementation, the coupling loss of Si_3_N_4_ waveguides with fibers can be much less than 1 dB/facet [36, 37]. The coupling loss of 2.5 dB/facet posed a limit on the CE of our current nonlinear Si_3_N_4_ waveguides which were manually cleaved. One-to-two-millimiter-long 300-nm-wide lossy single-mode Si_3_N_4_ waveguides were left as buffer between the tapers and facets after the manual cleaving, which contributed to the large coupling loss. By improving the waveguide coupling via optimized design and fabrication such as deep etching for facet generation, we expect that the CE will increase.

## 4 Conclusions

Flexible 100-Gbps-beyond all-optical WC of single-polarization coherent signals with a high-order modulation format is successfully demonstrated for the first time without any optical amplification in the signal path, using a low-loss dispersion-engineered Si_3_N_4_ nonlinear nanophotonic waveguide which is back-end CMOS compatible. Optical spectral translation from S band to C band as well as multichannel WC in C band is achieved, based on the same *χ*^(3)^ integrated waveguide. The high pump-flexible on-chip/fiber-to-fiber CEs up to −0.6/−5.6 dB are strong advantages of FWM-based optical signal processing. Besides, the WC bandwidth can be dramatically expanded, and the CE can be further increased via small feasible modifications of the current Si_3_N_4_ waveguide. Hence, the high-efficiency WC based on the low-loss nonlinear Si_3_N_4_ nanophotonic platform is promising for flexible spectral translation of wideband signals in optical fiber communications and may find applications in many other areas such as free-space optical communications [38], microwave photonics [39], metrology [40], optical imaging [41, 42], optical signal processing [43], and quantum optics [44].
